# Development and evaluation of an outbreak surveillance system integrating whole genome sequencing data for non-typhoidal *Salmonella* in London and South East of England, 2016–17

**DOI:** 10.1017/S0950268821001400

**Published:** 2021-06-28

**Authors:** Karthik Paranthaman, Piers Mook, Daniele Curtis, Edward-Wynne Evans, Emma Crawley-Boevey, Girija Dabke, Kevin Carroll, Jacquelyn McCormick, Timothy J. Dallman, Paul Crook

**Affiliations:** 1 Field Service, National Infection Service, Public Health England, London, UK; 2 Warwick Medical School, University of Warwick, Coventry, UK; 3 PHE London, Public Health England, London, UK; 4 PHE South East, Public Health England, Fareham, UK; 5 Gastrointestinal Pathogens Unit, National Infection Service, Public Health England, London, UK; 6 Gastrointestinal Bacteria Reference Unit, National Infection Service, Public Health England, London, UK

**Keywords:** Epidemiology, gastrointestinal, *Salmonella*

## Abstract

An outbreak surveillance system for *Salmonella* integrating whole genome sequencing (WGS) and epidemiological data was developed in South East and London in 2016–17 to assess local WGS clusters for triage and investigation. Cases genetically linked within a 5 single-nucleotide polymorphism (SNP) single linkage cluster were assessed using a set of locally agreed thresholds based on time, person and place, for reporting to local health protection teams (HPTs). Between September 2016 and September 2017, 230 unique 5-SNP clusters (442 weekly reports) of non-typhoidal *Salmonella* 5-SNP WGS clusters were identified, of which 208 unique 5-SNP clusters (316 weekly reports) were not reported to the HPTs. In the remaining 22 unique clusters (126 weekly clusters) reported to HPTs, nine were known active outbreak investigations, seven were below locally agreed thresholds and six exceeded local thresholds. A common source or vehicle was identified in four of six clusters that exceeded locally agreed thresholds. This work demonstrates that a threshold-based surveillance system, taking into account time, place and genetic relatedness, is feasible and effective in directing the use of local public health resources for risk assessment and investigation of non-typhoidal *Salmonella* clusters.

## Introduction

Non-typhoidal *Salmonella* infections are responsible for a considerable burden of morbidity and mortality in both developing and developed countries [1]. As such, a core responsibility of public health agencies is to ensure robust surveillance systems for rapid detection and investigation of *Salmonella* outbreaks. Methods for outbreak detection include indicator-based systems using routinely collected case data and event-based systems utilising data from any source [2]. Laboratory surveillance systems complement traditional epidemiological methods by allowing delineation of outbreak cases from unrelated cases [3].

Whole genome sequencing (WGS) is increasingly being used as the routine method for molecular characterisation of gastrointestinal (GI) pathogens in national reference laboratories [4, 5]. Application of WGS supports more robust case ascertainment and comparison of food and environmental samples with human samples [6, 7]. Nevertheless, the use of WGS data to inform real-time surveillance to identify outbreaks poses a number of challenges. These include lack of consensus on methods for analysis of WGS data and the difficulties in integrating genetic and epidemiological information to identify suspected outbreaks [8, 9]. Cases previously categorised by less discriminatory typing systems as apparent sporadic cases may be more linked genetically by the unprecedented resolution of WGS, thus leading to an increase in the number of potential outbreaks detected. Since April 2014, all presumptive *Salmonella* sp isolates received at the Gastrointestinal Bacteria Reference Unit (GBRU) of Public Health England (PHE) have been sequenced. A review of WGS data for the period April 2016 to March 2018 found that a large proportion of *Salmonella* WGS clusters in England were small, with fewer than five cases [5].

In epidemiology, the term cluster is used for cases that are linked in time and place but without a known epidemiological link. The term outbreak is used when there is a clear excess of cases compared to the baseline or an epidemiological link is confirmed in terms of common exposure(s) between the cases. In this report, the term cluster is used to indicate isolates that are genetically related by WGS but may or may not be linked in person, place and time. In practical terms, detection of an epidemiological or WGS cluster implies the need for further investigation to elucidate the transmission pathway from source to cases, in order to inform effective control measures. In the context of routine sequencing of all isolates, genetic relatedness as quantified by WGS data can be integrated with time, space and person attributes in defining clusters [9]. The increasing numbers of clusters detected as a consequence of routine WGS are likely to exceed the capacity of public health agencies to investigate, requiring the need for triaging and targeted epidemiological investigations. Attempts have been made to assess the likely operational burden generated by a WGS-based surveillance system for non-typhoidal *Salmonella* in England with cluster definitions based on varying genetic and epidemiological levels [10].

In early 2016, we developed and implemented a new WGS cluster surveillance system to enable rapid risk assessment and public health response to local as well as national clusters. In this paper, we present the considerations in the development of the system utilising WGS data for non-typhoidal *Salmonella* in London and the South East of England and the public health value of this system in the prioritisation and investigation of clustered cases.

## Methods

### Organisational arrangements

PHE's Gastrointestinal Pathogens Unit (GPU) is responsible for national surveillance of GI infections and the detection of and response to national outbreaks. PHE's Health Protection Teams (HPTs) based within nine PHE centres are responsible for leading the public health response to local outbreaks constrained to their catchment populations and for supporting investigations of national outbreaks affecting multiple PHE centres. PHE's Field Service (FS) is a nationally coordinated but geographically dispersed service with a role in supporting field epidemiological investigations and surveillance of infectious diseases, both at the local and national level. The FS team covering South East and London (SEaL) provides field epidemiology support to seven HPTs in London and the South East with an estimated population of 17 905 826 in 2017 [11]. The national reference laboratory GBRU is responsible for confirmation and characterisation of *Salmonella* isolates submitted by frontline testing laboratories.

### Laboratory surveillance methods

Frontline diagnostic laboratories in England are legally required to report confirmed *Salmonella* cases to PHE. Referral of locally confirmed *Salmonella* isolates to GBRU is strongly encouraged yet remains voluntary. It is estimated that the GBRU receives approximately 95% of all isolates of *Salmonella* detected at diagnostic laboratories. Approximately 8000 human isolates are referred per year in England, of which just over a third are reported from the South East and London [12]. A comprehensive description of reference laboratory methods employed at GBRU and surveillance for *Salmonella* in England has been published elsewhere [5].

### WGS cluster analysis

Following sequencing, single-nucleotide polymorphism (SNP) analysis is the primary method in England for assessing the genetic relationship between isolates, allowing comparison of a seven number ‘SNP address’ given for each isolate [13]. SNP analysis is undertaken for the most commonly reported eBURST groups (eBGs) and Sequence Types (STs) with coverage estimated at 85% of sequenced isolates during 2016–17 [5, 13]. Data relating to each isolate including case demographic details and SNP addresses are held in Gastro Data Warehouse (GDW), a dedicated national database. For *Salmonella*, previous validation studies have proposed that cases whose isolates cluster together using a 5-SNP threshold within a single linkage methodology are likely to share a common source of infection [9]. PHE defines a *Salmonella* 5-SNP cluster as comprising at least two human cases within a 5-SNP single linkage cluster, corresponding to a maximum of 5 SNP between two adjacent isolates, not between the most distant isolates of the cluster. A ‘new cluster’ is defined as one where at least two human isolates are within a 5-SNP cluster and this is the first week there have been at least two cases with that SNP address and all isolates in the new cluster are more than 5-SNPs away from existing *Salmonella* isolates. An ‘active cluster’ is defined as one where newly sequenced isolates are within 5 SNPs of at least one isolate falling into a previously identified 5-SNP cluster. The GPU produce and disseminate to FS teams and HPTs a weekly summary (‘SNP cluster tool’) with details of all WGS clusters identified in the previous week among residents in England. The SNP cluster tool presents summary statistics including cluster size, cluster age and cluster growth rate on new and active 5-SNP clusters to which at least one new isolate (either human, food, water, animal or environmental) has been added in the last 8 days to GDW. ‘Cluster size’ refers to the number of cases in the cluster at the designated 5-SNP level. ‘Cluster age’ is the time period in months between the specimen date for the index case in the cluster and the most recently detected case. ‘Cluster growth rate’ is the cluster size divided by the cluster age in months, rounded to one decimal point.

### Development of local threshold-based surveillance and reporting process

In early 2016, the FS SEaL team and local HPTs agreed on the need for a local threshold-based surveillance system for reporting WGS clusters with cases resident in the South East or London. The surveillance system aimed to identify new cases belonging to known and active outbreak investigations to facilitate rapid follow-up, and WGS clusters with cases exceeding locally agreed thresholds requiring local risk assessment and public health response. In addition, WGS clusters with cases below locally agreed thresholds may be reported for monitoring purposes.

**Fig. 1.**
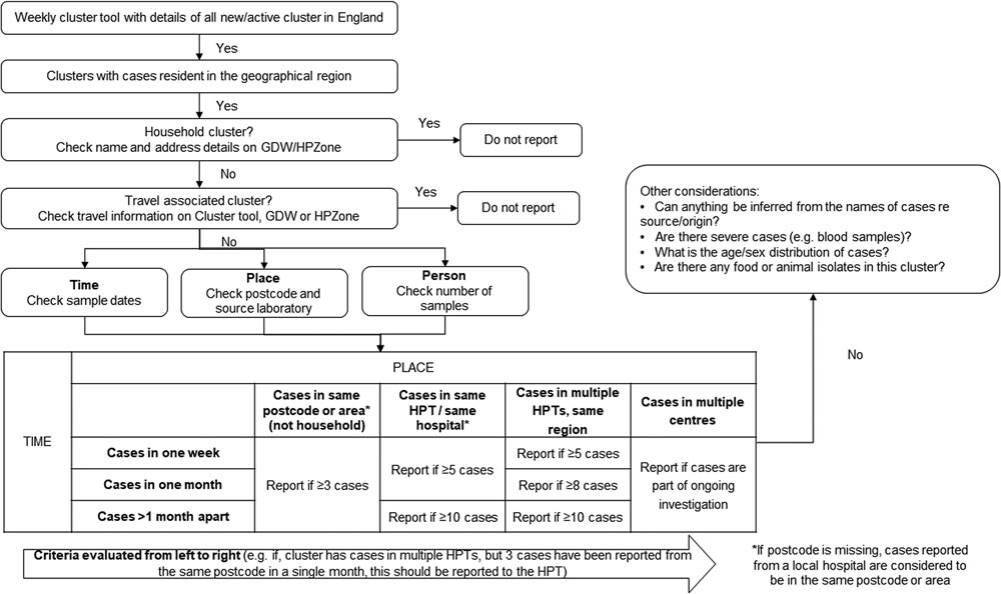
Flowchart to assess reporting of non-typhoidal *Salmonella* WGS clusters, South East and London, 2016–17.

Case thresholds for local reporting of WGS clusters (Fig. 1) were based on cluster characteristics such as location (geographic distribution), size (number of cases), duration (cases over time) and travel history (reported foreign travel). These thresholds were informed by published scientific literature, expert advice on the application of WGS data for local surveillance, and local senior staff judgement on resource implications and public health benefit of investigating such clusters. The agreed thresholds were chosen explicitly to identify a rapidly growing cluster of cases in a small geography within a tight timeframe (indicative of point-source outbreak) as well as more slow growing, larger clusters spanning a longer time span or larger geographical catchment (indicative of seasonal or continuous transmission).

Household clusters were excluded from local reporting, as HPTs were often aware of the epidemiological link in the course of routine public health response to known *Salmonella* cases, before the receipt of cluster information in the weekly SNP cluster tool. Foreign travel-associated clusters, as assessed by the GPU to be likely associated with travel abroad to a specified location/region in the incubation period, were excluded from local reporting to HPTs because they were deemed to be unlikely to lead to local public health interventions.

Additional factors considered in the assessment included unusual patterns in age-sex distribution, ethnicities/origin as inferred by patient names, severity of illness and presence of a related food/environmental sample but these factors were primarily used for hypothesis generation of source(s)/vehicle(s) and not used as a sole criterion for reporting.

Trained staff in FS SEaL team reviewed the weekly SNP cluster tool and reported relevant clusters to the local HPTs through a weekly cluster report notification by email. A decision log was maintained by the FS SEaL team of all clusters assessed and reported weekly to local HPTs. As clusters often grow over time, the same cluster could be reported to HPTs several times with updated numbers of cases. In case of unusual clusters where decision-making on risk assessment and response was not straight forward (e.g. slow growing clusters that were just under-reporting criteria or with additional cases dispersed in areas outside the local catchment), FS SEaL staff discussed the cluster epidemiology and management with relevant colleagues from the GPU, GBRU and the HPT. Alongside the development of guidance materials on the science and operational processes, training workshops were held to ensure that HPT staff understood the strengths and limitations of the surveillance system.

### Evaluation of local threshold-based surveillance system

In early 2018, a review of all WGS clusters assessed by FS SEaL between 5 September 2016 (iso-week 36) and 11 September 2017 (iso-week 37) was undertaken. As part of this evaluation, details on HPT investigations and management of reported clusters during the above period were collected using a standardised proforma. Additional information on reported clusters was also extracted from HPZone, a dedicated case management system where HPTs record details of public health management of cases and outbreaks.

For the purpose of analysis, 5-SNP clusters with cases in multiple HPTs that were reported on several occasions during the study period were deduplicated and reported as ‘unique’ 5-SNP clusters, disregarding any changes in SNP addresses to a small number of clusters that merged within the study period. A cluster investigation was deemed to be active if there was evidence in the standardised proforma or in HPZone that an incident management team was convened or that the HPT or national team were seeking exposure data from questionnaires for clustered cases. Cluster-related characteristics (such as size, geographical and temporal distribution, age, outcome) were analysed and reported descriptively using R [14]. The ‘boot.ci’ function in ‘boot’ package was used to calculate two-sided non-parametric confidence intervals (with normal approximation) for medians for clusters with 10 000 resamples for each category of reporting.

## Results

Between 5 September 2016 (iso-week 36) and 11 September 2017 (iso-week 37), 230 unique 5-SNP clusters (442 weekly reports) of non-typhoidal *Salmonella* WGS clusters were reported among residents in London and South East. The range for all 442 clusters in the study period was 2–345 cases. The median growth rate was 1.7 and the median cluster age was 14 months. *Salmonella* Enteritidis and Typhimurium accounted for over 80% of all clusters reported during the study period. Key characteristics of all clusters reported in the study period by serovar are provided in Table 1.

**Table 1.** Characteristics of non-typhoidal *Salmonella* WGS clusters by serovar, South East and London, September 2016–2017

Serovar	Number of weekly reports	Number of unique SNP clusters	Median cluster size^a^	Minimum cluster size^a^	Maximum cluster size^a^	Median cluster age^a^	Median cluster growth^a^
Anatum	1	1	2	2	2	7	0.3
Bovismorbificans	1	1	2	2	2	32	0.1
Braenderup	5	4	2	2	3	0	3.4
Stanley	5	4	2	2	6	0.1	15.4
Brandenburg	1	1	3	3	3	0.6	5.4
Infantis	7	7	3	2	13	0	0.6
Virchow	2	1	3.5	3	4	0.2	10.4
Kentucky	7	5	4	2	20	7	0.6
Newport	17	9	4	2	10	0.4	2.3
Oranienburg	1	1	4	4	4	26	0.1
Typhimurium	149	85	5	2	133	0	1.5
Agona	14	8	6.5	2	59	1	1.1
Mikawasima	2	2	9	8	10	0.5	11.2
Chester	3	1	14	4	18	4	2.8
Enteritidis	227	100	15	2	345	0	2

a Indicates median, minimum or maximum value if more than one cluster reported in each category during study period.

At the end of the study period, 208 unique 5-SNP clusters (316 weekly reports) did not fulfil the agreed thresholds for reporting and hence were not reported to HPTs; the remaining 22 unique clusters (126 weekly clusters) were reported to HPTs (*P* < 0.0001, *χ*^2^ test). The median cluster age of weekly clusters for those reported to the HPTs tended to be higher (21 *vs.* 10.5 months), compared to those that were not reported.

**Fig. 2.**
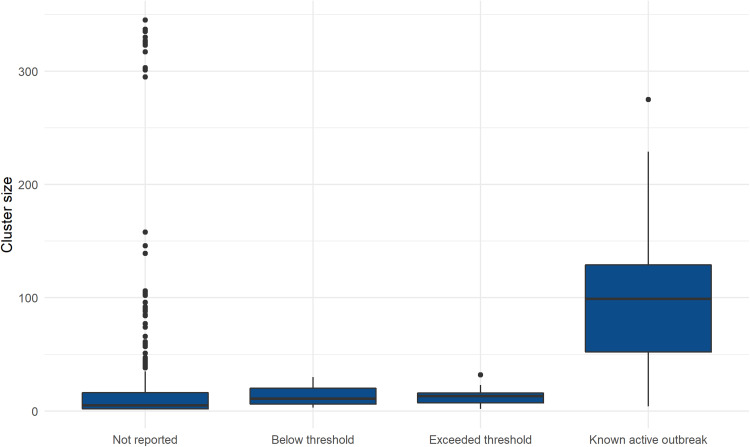
Distribution of median cluster sizes of non-typhoidal *Salmonella* WGS clusters by reason for reporting to HPTs, South East and London, September 2016–2017.

**Fig. 3.**
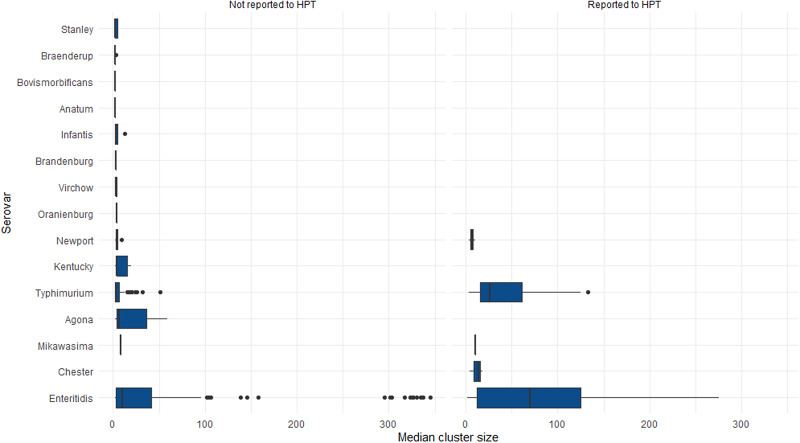
Distribution of median cluster sizes of non-typhoidal *Salmonella* WGS clusters by serovar and report status to HPT, South East and London, September 2016–2017.

Of the unique clusters reported to HPTs, nine were known active outbreak investigations, seven were below locally agreed thresholds and six exceeded local thresholds. Characteristics of clusters by reason for reporting to HPTs are provided in Table 2. The distribution of median cluster sizes by reason of reporting to HPTs and serovar is provided in Figures 2 and 3 respectively. The median cluster size for those clusters not reported to HPTs was five cases (95% confidence interval (CI) 4.3–6.5) and 27 cases (95% CI 10.9–40.2) for clusters reported to HPTs. Compared to all other clusters, the median cluster size was largest for clusters that were known and under active investigation (*P* < 0.0001, Wilcoxon rank-sum test). Among serovars, *S.* Enteritidis clusters had the highest median case numbers.

**Table 2.** Characteristics of non-typhoidal *Salmonella* WGS clusters by reason for reporting to HPTs, South East and London, September 2016–2017

Report to HPT	Reason for report	Number of weekly reports	Number of unique SNP clusters	Median cluster size^a^ (confidence intervals)^b^	Minimum cluster size^a^	Maximum cluster size^a^	Median cluster age^a^	Median cluster growth^a^
No	Not reported	316	208	5 (4.3–6.5)	2	345	10.5	1.1
Yes	Below threshold	33	7	11 (5.1–16.2)	3	30	3	2
	Above threshold	27	6	13 (9.9–16.6)	2	32	6.5	2.8
	Known active outbreak	66	9	99 (83.6–116.8)	4	275	31.5	3.2

a Indicates median, minimum or maximum value if more than one cluster identified in each category during study period.

b Calculated by bootstrapping.

Among the 22 unique clusters reported to HPTs, nine unique clusters were reported as part of known active outbreak investigations (Table 3). Of note, three *Salmonella* Enteritidis 5-SNP clusters nested within a 25-SNP cluster were linked to a common source and hence were managed as a single outbreak investigation [15]. Two *Salmonella* Enteritidis 5-SNP clusters nested within a 50-SNP cluster were linked to a common source/vehicle [6]. A source and/or vehicle was known for all nine 5-SNP clusters that were known and under active investigation. For the majority of these clusters, the median cluster age was between 20 and 30 months, indicating sustained transmission. Geographical distribution varied between extremes of tight local clusters restricted to one region to nationally dispersed clusters across several regions.

**Table 3.** Characteristics of non-typhoidal *Salmonella* WGS clusters (*n* = 9) that were known active outbreaks and reported to HPTs, South East and London, September 2016–2017

Serovar	Number of weekly reports	Median cluster size^a^	Minimum cluster size^a^	Maximum cluster size^a^	Median cluster age^a^	Median cluster growth^a^	Geographic distribution	Source/vehicle/setting
Typhimurium	1	5	5	5	22	0.2	1 region (South East)	Outbreak in a farm; cattle on farm unwell and positive for *Salmonella* but not sequenced
Enteritidis	3	9	8	14	24	0.4	1 region (South East)	Three WGS linked cases among at least 23 symptomatic cases in a children's nursery detected by traditional methods
Chester	3	14	4	18	5	2.8	7 regions and 2 DAs^b^	Stir fry products
Enteritidis	3	50	39	52	34	1.5	9 regions and 4 DAs	Two different 5-SNP clusters linked to the same source (eggs) from Poland [6]
	14	95.5	52	140	33.5	2.8		
Typhimurium	18	69	31	133	20.5	3.4	8 regions and 1 DA	Livestock in the UK
Enteritidis	10	107.5	70	126	36	3	9 regions and 3 DAs	Three 5-SNP clusters linked to eggs and chicken products from Spain [15]
	12	161	132	189	34.5	4.6		
	2	252	229	275	32.5	7.7		

a Indicates median, minimum or maximum value if more than one cluster identified in each category during study period.

b Devolved administration.

In contrast, a source/vehicle was not identified in any of the seven clusters that did not breach agreed thresholds (Table 4). Median cluster age for this category tended to be lower compared to those known and under investigation and there were no defining patterns of geographical distribution.

**Table 4.** Characteristics of non-typhoidal *Salmonella* WGS clusters (*n* = 7) below local thresholds and reported to HPTs, South East and London, September 2016–2017

Serovar	Number of weekly reports	Median cluster size^a^	Minimum cluster size^a^	Maximum cluster size^a^	Median cluster age^a^	Median cluster growth^a^	Geographical distribution	Source/vehicle
Enteritidis	3	4	3	5	2	1.7	1 region (London)	Not investigated
Enteritidis	6	6.5	4	12	2.5	2.7	4 regions	Not investigated
Newport	6	6.5	3	10	3	2	3 regions	Not investigated
Typhimurium	1	7	7	7	27	0.3	1 region (South East)	Not investigated
Mikawasima	1	10	10	10	0.5	21.7	4 regions and 1 DA^b^	Questionnaires sought but not received
Typhimurium	9	20	15	26	25	0.8	3 regions	Questionnaires sought but not received
Typhimurium	7	23	5	30	2	13.7	6 regions and 1 DA	Not investigated

a Indicates median, minimum or maximum value if more than one cluster identified in each category during study period.

b Devolved administration.

For the last category of six clusters that exceeded local thresholds, the cluster sizes were small and geographic distribution was more mixed. Nevertheless, a source or vehicle was identified in four of six clusters (all with over 10 cases). Characteristics and outcomes of these clusters are summarised in Table 5.

**Table 5.** Characteristics of non-typhoidal *Salmonella* WGS clusters (*n* = 6) that exceeded local thresholds and reported to HPTs, South East and London, September 2016–2017

Serovar	Criteria exceeded	Number of weekly reports	Median cluster size^a^	Minimum cluster size^a^	Maximum cluster size^a^	Median cluster age^a^	Median cluster growth^a^	Geographical distribution	Source/vehicle
Typhimurium	⩾3 cases in same postcode/area in 1 week	1	5	5	5	22	0.2	1 region (South East) and 2 DAs^b^	Not identified
Enteritidis	⩾3 cases in same postcode/area in 1 week	6	11	4	14	1	13.5	5 regions	Not identified
Enteritidis	⩾5 cases in same HPT in 1 month	7	12.5	2	19	15	0.8	4 regions	Eggs from a specific flock in England [16]
Enteritidis	⩾8 cases in multiple HPTs, same centre in 1 month	3	13	5	18	2	6	5 regions	Egg mix and Arancini at a local food establishment
Typhimurium	⩾10 cases in multiple HPTs, same PHE centre in 1 month	7	14	3	32	7	2	7 regions	Lamb/sheep in England [17, 18]
Enteritidis	⩾5 cases in same HPT in 1 month	3	16	14	18	5	3.2	3 regions and 1 DA	Linked to a local food establishment but no food samples available

a Indicates median, minimum or maximum value if more than one cluster identified in each category during study period.

b Devolved administration.

## Discussion

Despite the well-proven application of WGS in non-typhoidal *Salmonella* outbreaks, there is little published literature on the integration of WGS data in routine *Salmonella* outbreak surveillance systems and public health response [8, 9]. The work presented in this paper describes the development of a local threshold-based surveillance system in the South East of England and London integrating WGS and epidemiological data for non-typhoidal *Salmonella* and highlights the outcomes for reported clusters following public health investigation.

The introduction of comprehensive routine sequencing of all referred *Salmonella* isolates at the reference laboratory in England has resulted in a step change in PHE's ability to detect outbreaks [5]. Data on genetic relatedness, hitherto unavailable, has now been integrated to ‘retune the surveillance radar’ to allow more sensitive detection of outbreaks. As WGS offers very high strain discrimination capability, a more robust delineation of outbreak cases from non-outbreak cases is now a reality. This has enhanced the effectiveness of targeted epidemiological investigations as there is increased confidence that cases clustered by WGS are more likely to share exposure to a common contaminated environment, animal or food source. Furthermore, investigations to detect the source are much less likely to be biased by the inclusion of cases that are not part of the outbreak. In addition, the degree of genetic relatedness and ancestry based on phylogenetic data could be used to hypothesise likely exposure to a common source [9].

During the evaluation phase in 2016–17, only a minority of clusters (six of 22) reported to HPTs breached the locally agreed thresholds. Among these six clusters, four led to successful identification of the vehicle/source and implementation of control measures. Integration of WGS data into the outbreak surveillance system was instrumental in the identification of these four outbreaks. In the counterfactual scenario, we consider that traditional surveillance systems alone, in the absence of WGS data, would not have identified the smaller outbreaks and substantially delayed the identification of the larger outbreaks. However, there are valid grounds for criticism that the agreed thresholds were set too high. Insofar as public health capacity for investigation is constrained, it must be acknowledged that there exists no single ‘right’ threshold level for intervention and a judgement has to be made by those in senior positions to achieve the balance between optimising the use of public resources while ensuring the safety of public health.

For those clusters where the investigation was successful in identifying source/vehicle, control measures were implemented as appropriate to the source/vehicle and setting involved. These varied from inspection of premises linked to transmission (e.g. food business, nursery, etc.), enhanced cleaning and hygiene measures at specific venues, withdrawal of implicated products and provision of appropriate management information to those at risk as well as healthcare professionals.

In the remaining 13 clusters, nine were known active national or local outbreaks and seven with cases below agreed thresholds were reported on the basis of professional judgement of FS SEaL staff. Active investigations were attempted in two of the seven clusters with cases below agreed thresholds, but none resulted in the identification of a common source/vehicle. The rationale for alerting HPTs of these clusters was to allow monitoring of the cluster and to conduct a rapid risk assessment where appropriate.

It is important to note that of the 230 unique clusters assessed, 208 clusters did not fulfil thresholds for reporting and therefore did not trigger further local investigations. As *Salmonella* exposure questionnaires are not routinely sought from cases in the local catchment regions, no further details on these clusters were available. To be able to identify common exposures for small genetically linked temporo-spatial clusters, the challenge for epidemiologists and public health agencies is to strengthen processes to routinely collect detailed epidemiological data more promptly and efficiently than is the case currently.

Exclusion of household clusters and travel-associated clusters reduced the burden of reporting and investigation at the local level. Household clusters are often known to HPTs as part of standard case management. In addition, they tend to be small clusters and pose little public health risk outside of the household. Foreign travel-associated clusters were not reported to local HPTs because there is a clearly defined process for PHE's national travel health team to liaise with relevant international agencies as required. Among the 442 clusters in the study period, four travel-associated clusters were identified but not reported to local HPTs.

The majority of WGS clusters during the study period belonged to serovars *S*. Typhimurium and *S*. Enteritidis, reflecting the epidemiology of *Salmonella* in England [12]. Known outbreaks under active investigation were much larger in size compared to other WGS clusters. These large outbreaks highlight stubborn challenges in either identifying the source/vehicle or putting in place effective control measures. Of note, two large outbreaks had three and two 5-SNP clusters nested within a 25 and 50-SNP cluster but linkage within a larger genetic cluster was secondary to the finding that the individual 5-SNP clusters were linked to a common source in both outbreaks [6, 15]. In other words, these two outbreaks do not provide any evidence to expand the 5-SNP threshold for outbreak detection [5]. Persistent, large outbreaks that are sustained over a longer period reflect their continued public health impact and should lead to redoubling of efforts to quantify the burden of disease as well as recognising any changes in transmission pathways to achieve effective control. In contrast, rapidly growing small to moderate sized outbreaks signal the onset of a new public health risk (e.g. contaminated food product or environmental exposure), which if investigated robustly may be amenable to prompt control.

This surveillance system was designed together by staff with expertise in genomics, bioinformatics, epidemiology, surveillance and infectious disease control. In particular, PHE has developed a secure data infrastructure to allow rapid analysis of WGS data [13]. Production of the weekly SNP cluster tool has been automated in software R [14]. Work to automate the process of reviewing the weekly SNP cluster tool to identify local clusters that fulfil agreed thresholds has been delayed due to the ongoing Covid pandemic but is expected to be prioritised in the next few months. In addition to holding regular training events on WGS clusters, staff also have rapid, reliable access to expert advice on the interpretation of WGS data and risk assessment of complex clusters. We attempted to quantify the resource implications at the local level for this surveillance system. However, this was unsuccessful because of the differences in how different HPTs managed the process locally. In brief, the requirements are that HPT staff review the weekly WGS cluster report emails and undertake further investigations if a cluster warranted it.

There are several limitations to this work. First, the thresholds agreed for identifying and reporting clusters were agreed locally based on scientific evidence as well as resource considerations. Smaller clusters that did not breach the agreed thresholds were not actively investigated by HPTs. If unlimited resources were available, active investigation of all clustered cases could have led to the identification of additional public health risks amenable for remediation. Given the inevitable budgetary constraints on public agencies, we consider that the current approach represents a proportionate strategy for prioritisation and investigation of *Salmonella* clusters that pose a public health risk. Second, there could have been some differences in the way trained staff applied judgement in evaluating and reporting clusters that did not meet the thresholds and this was not formally assessed in the evaluation. Third, attempts were made to quantify the resource implications at the HPT level in responding to this surveillance system. However, in view of substantial variation in local processes and lack of standardisation, it could not be interpreted.

Our work demonstrates that an integrated threshold-based surveillance system, taking into account time, place and genetic relatedness, is feasible and effective in directing the use of public health resources for risk assessment and investigation of non-typhoidal *Salmonella* clusters. This initiative is underpinned by strong collaboration between microbiologists, bioinformaticians, epidemiologists and local health protection staff with robust arrangements for data sharing, communication and access to expert advice.

## Data

We are unable to share the data used in this study due to confidentiality and legal reasons.
